# Changes in the Frontotemporal Cortex and Cognitive Correlates in First-Episode Psychosis

**DOI:** 10.1016/j.biopsych.2010.03.019

**Published:** 2010-07-01

**Authors:** Leticia Gutiérrez-Galve, Claudia A.M. Wheeler-Kingshott, Daniel R. Altmann, Gary Price, Elvina M. Chu, Verity C. Leeson, Antonio Lobo, Gareth J. Barker, Thomas R.E. Barnes, Eileen M. Joyce, María A. Ron

**Affiliations:** a University College London Institute of Neurology, London, United Kingdom; b Hospital Clínico Universitario and Universidad de Zaragoza, Centro de Investigación Biomédica en Red de Salud Mental and Instituto Aragonés de Ciencias de la Salud, Zaragoza, Spain; c Medical Statistics Unit, London School of Hygiene and Tropical Medicine, London, United Kingdom; d King's College London, Institute of Psychiatry, Department of Clinical Neuroscience, Centre for Neuroimaging Sciences, London, United Kingdom; e Imperial College Faculty of Medicine, Charing Cross Campus, London, United Kingdom

**Keywords:** Cognitive impairment, cortical area and thickness, first-episode psychosis, frontotemporal cortex, magnetic resonance imaging, surface-based morphometry

## Abstract

**Background:**

Loss of cortical volume in frontotemporal regions has been reported in patients with schizophrenia and their relatives. Cortical area and thickness are determined by different genetic processes, and measuring these parameters separately may clarify disturbances in corticogenesis relevant to schizophrenia. Our study also explored clinical and cognitive correlates of these parameters.

**Methods:**

Thirty-seven patients with first-episode psychosis (34 schizophrenia, 3 schizoaffective disorder) and 38 healthy control subjects matched for age and sex took part in the study. Imaging was performed on an magnetic resonance imaging 1.5-T scanner. Area and thickness of the frontotemporal cortex were measured using a surface-based morphometry method (Freesurfer). All subjects underwent neuropsychologic testing that included measures of premorbid and current IQ, working and verbal memory, and executive function.

**Results:**

Reductions in cortical area, more marked in the temporal cortex, were present in patients. Overall frontotemporal cortical thickness did not differ between groups, although regional thinning of the right superior temporal region was observed in patients. There was a significant association of both premorbid IQ and IQ at disease onset with area, but not thickness, of the frontotemporal cortex, and working memory span was associated with area of the frontal cortex. These associations remained significant when only patients with schizophrenia were considered.

**Conclusions:**

Our results suggest an early disruption of corticogenesis in schizophrenia, although the effect of subsequent environmental factors cannot be excluded. In addition, cortical abnormalities are subject to regional variations and differ from those present in neurodegenerative diseases.

Cortical volume is genetically determined with heritability (1) decreasing over time as environmental factors become relevant (2). The cortex is shaped in utero by the strength of interregional connectivity, and cortical changes are to be expected in schizophrenia, a disease with abnormal brain connectivity (3,4).

Meta-analyses of voxel-based morphometry studies identified gray matter loss in corticosubcortical networks involving frontotemporal and limbic cortex, thalamus, and striatum in chronic schizophrenia (5–7), and less extensive changes have also been reported in those with first-episode (8), schizotypal disorder, and high-risk individuals (9–12). Subtle cortical abnormalities, without volume loss, have been described using magnetization transfer imaging in first-episode psychosis (13,14).

Surface-based morphometry (SBM) methods allow the independent measurement of cortical area and thickness—indexes that share a high heritability but are determined by different genetic mechanisms (15). Freesurfer (16), an SBM method with realistic cortical reconstruction that allows for manual correction of topological errors (17), uses a segmentation procedure based on the identification of gray–white matter and gray matter–pial boundaries, as well as a surface-based registration that aligns cortical folding patterns. Freesurfer has an accuracy of .2 mm (18) compared with postmortem measures of cortical thickness and has been validated using different scanners and magnetic resonance imaging (MRI) protocols (16).

Studies using SBM have mainly measured regional or whole cortical thickness. Frontotemporal gyral and sulcal thinning has been reported in children and adolescents (19,20) and in young adults with first-episode schizophrenia by some (21,22) but not others (23). Cortical thinning, particularly prefrontal, followed a developmental trajectory different from that of control subjects (24,25). In chronic patients, thinning of the entorhinal (26) and frontotemporal cortex with relative sparing of somatosensory (27) and parietal cortex (28) has also been reported and changes in cortical thickness in the cingulate and temporal regions may represent the unexpressed genetic liability in relatives of schizophrenia patients (29).

Cortical area has been less frequently measured with contradictory results. Thus, although reduced area in paralimbic ventral frontal cortex in drug-naive patients (30) and in posterior cingulate in those with chronic schizophrenia and their relatives has been described (31), other studies (32) have reported area increases in the anterior cingulate. Early reports of decreased insular area in first-episode patients (30) have not been replicated (33). A study of patients with adolescent-onset schizophrenia (34) identified widespread, regionally variable cortical pathology in prefrontal and superior temporal gyrus, with variable reductions in area and/or thickness. Similar changes have been reported in unaffected relatives (35), and severity of positive symptoms has been associated with decrease entorhinal area (26).

We report here an exploratory study in patients with first-episode psychosis in whom cortical area and thickness were measured using SBM in frontal and temporal cortex, regions known to be implicated in schizophrenia. We aimed to clarify the pattern of cortical abnormalities, whether changes in area and cortical thickness occurred independently, and their possible associations with clinical and cognitive measures.

## Methods and Materials

### Subjects

Patients were part of a cohort recruited for the West London Longitudinal First-Episode Psychosis Study (36), aged between 16 and 49 years at recruitment, who had been receiving antipsychotic medication for less than 12 weeks. Diagnosis was ascertained using the diagnostic module of the Diagnostic Interview for Psychosis (DIP-DM) (37), which includes items from the Operational Criteria Checklist for Psychosis (38) and the World Health Organization Schedules for Clinical Assessment in Neuropsychiatry (39). Two nurses trained by an experienced psychiatrist (T.R.E.B.) conducted the interviews.

Forty-one patients who had MRI and neuropsychologic assessments participated. Four were excluded because of poor-quality MRI data. Thirty-seven patients (25 males) were included; 34 had a final diagnosis of schizophrenia and 3 had schizoaffective disorder (1 bipolar, 2 depressed subtypes). Mean age was 26.8 years (SD = 8.8; range, 16–49). The study was naturalistic with no restrictions on prescribed medication; all patients were prescribed antipsychotics (36 second-generation, 1 first-generation), and 8 were also prescribed antidepressants. At the time of scanning, the median duration of treatment was 102 days (range, 0−384), and 17 patients had received treatment for more than 12 weeks. Thirty-eight healthy subjects (22 males) who had neuropsychologic assessment and MRI served as control subjects. Their mean age was 25.0 years (SD = 5.4; range, 16–37). Exclusion criterion for all subjects, was the presence of a medical or neurological illness, including head injury leading to unconsciousness. Controls with psychiatric illness in themselves or their first-degree relatives were excluded.

### Clinical Ratings

Mental state was assessed with the Scales for the Assessment of Positive and Negative Symptoms (SAPS and SANS) (40,41). Interrater agreement (linearly weighted kappa) was assessed using a standard set of videotaped interviews. Using global subscale items, linearly weighted kappas of .76 for SAPS and .74 for SANS were achieved (42). Scores for the three symptom-derived syndromes (negative, positive, and disorganization) were calculated (43). Affective symptoms were measured with the Young Mania Scale (44) and Hamilton Rating Scale for Depression (45). Age of onset and duration of untreated psychosis (DUP) were established using the Symptom Onset in Schizophrenia Inventory (46). Alcohol and drug use were assessed using the DIP (37), and criteria for abuse and dependence using the Alcohol and Drug Use Scales (47). None of the subjects fulfilled these criteria. Handedness was assessed using the Annett Scale (48).

Ethical permission was obtained from the local ethics committees. Participants gave written informed consent according to the Declaration of Helsinki and received an honorarium.

### Neuropsychological Assessment

Premorbid IQ was estimated using the Revised National Adult Reading Test (49), validated in schizophrenia (50,51). Current IQ was measured using a short form of the Wechsler Adult Intelligence Scale—III (52) validated for schizophrenia (53), comprising the Information, Arithmetic, Block Design, and Digit Symbol subtests. Measures of executive function were derived from the Cambridge Neuropsychological Test Automated Battery (54): 1) *Working memory span*, from the Spatial Span Task, measures the ability to remember the order of sequences of colored squares presented in increasing numbers. The span was measured as the highest number recalled correctly. 2) *Working memory manipulation*, from the Spatial Working Memory Task, a self-ordered search task measures the ability to remember the location of previously found “tokens” while searching for new ones. An error occurs when a participant returns to the location where a token has already been found. Total errors were used as an index of working memory manipulation. 3) *Planning*, from the Stockings of Cambridge task (55), measures the ability to move colored “balls” in an arrangement displayed on the screen to match a goal arrangement. The number of problems solved in the minimum number of moves possible was the score.

Verbal memory was assessed with the Rey Auditory Verbal Learning Test (56). The participant is asked to recall nouns from a list of 15 immediately after each of five trials. The number of words recalled over the five trials was the final score.

### MRI Data Acquisition

The MRIs were performed on a GE Signa 1.5-Tesla scanner (General Electric, Milwaukee, Wisconsin), using a standard quadrature head coil. T1-weighted volumetric images were obtained using an inversion recovery spoiled gradient-recalled echo sequence with an isotropic voxel size of 1.2 × 1.2 × 1.2 mm^3^. One hundred twenty-four axial contiguous slices were acquired. Other parameters were echo time (5.4 msec), repetition time (15 msec), inversion time (450 msec), field of view = 31 × 16 cm^2^, acquisition matrix 256 × 128, number of averages = 1, excitation flip angle = 15°, and receiver bandwidth = 15.63 kHz.

### Image Processing

A rater (L.G.G.), blind to participant status, used Freesurfer 4.0.1 (http://surfer.nmr.mgh.harvard.edu) to generate maps of surface area and cortical thickness in standard Montreal Neurological Institute (MNI) space (57,58). After skull stripping and white matter segmentation, the cortical surface of each hemisphere was inflated to an average spherical surface to locate the pial surface and the gray–white matter boundary (57). The distance between the two at each vertex (i.e., surface point) across the cortex is considered a measure of cortical thickness. Cortical maps are smoothed with a 10-mm full-width at half-maximum Gaussian kernel and aligned to a common surface template using a high-resolution surface-based averaging technique, and 32 cortical parcellations are automatically generyated (59). The only manual step was the correction of topological errors when the above steps had been completed. Total brain volume was estimated using Freesurfer (60).

### Analysis of Cortical Parameters

Two comparisons were made between patients and controls: 1) whole-brain cortical thickness using the “vertex-by-vertex” analysis; and 2) cortical thickness, surface area, and volume in frontal and temporal regions.

**Figure 1:**
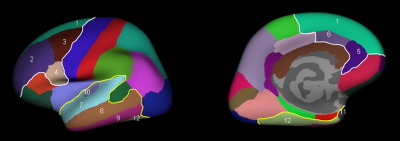
Lateral and midsagittal views of the frontal and temporal parcellations: 1, superior frontal; 2, rostral middle frontal; 3, caudal middle frontal; 4, pars opercularis; 5, rostral anterior cingulate; 6, caudal anterior cingulate; 7, superior temporal; 8, middle temporal; 9, inferior temporal; 10, transverse temporal; 11, temporal pole; 12, fusiform.

From the Desikan template (59), six frontal and six temporal parcellations in each hemisphere were selected. Superior frontal, pars opercularis, caudal middle frontal, rostral middle frontal, caudal anterior cingulate, and rostral anterior cingulate were selected in the frontal lobe; in the temporal lobe, the superior, middle, and inferior temporal, fusiform, temporal pole, and transverse temporal parcellations were selected (Figure 1). Average thickness, total surface area, and volume of the cortex for the frontal and temporal regions covered by these parcellations were calculated for each hemisphere and compared between the two groups.

### Statistical Analysis

#### Demographic and Cognitive Variables

Age, sex, total brain volume, and handedness were compared between groups using *t* and chi-square tests. Linear regression models adjusted by age and sex were used to compare cognitive scores.

#### Imaging Variables

Age and sex were used as covariates in all models.

For the comparison of whole brain cortical thickness, the vertex-by-vertex analysis was used, and means of cortical thickness were compared between groups using a two-tailed *t* test; cortical thickness was modeled as a function of group controlling for age and sex. Corrections for multiple comparisons were made using a false discovery rate (FDR), setting the level of significance at .05 (61).

Linear mixed models were used for the following comparisons of frontal and temporal cortical parameters between the groups: 1) differences due to diagnostic group, region, and side, with two-way interactions (diagnosis by region, diagnosis by side, and region by side); 2) differences due to sex; age; duration of treatment; DUP; and positive, negative, and disorganization syndrome scores with two-way interactions by region and side; and 3) associations between cognitive scores and cortical parameters with two- and three-way interactions. Region was entered as a within-subject effect and diagnosis, cognitive scores, sex, and age as between-subject effects. Three separate models were created for thickness, surface area, and cortical volume that allowed the inclusion of multiple measurements for each subject and the handling of missing data, thereby increasing statistical power (62). As in previous studies (27,34,63), we did not control for brain volume, a schizophrenia-related variable, because this would have obscured possible group differences.

We repeated the same model with two-way interaction (diagnosis by region) to identify cortical differences in each of the six frontal or temporal parcellations. When significant interactions were present, the linear mixed model was repeated with the corresponding indicators and interaction terms for the regional parcellations.

For these exploratory analyses significance was reported at the 5% level with no formal adjustment made for multiple comparisons, because this may be inappropriate (64,65) when no single null hypothesis covers the multiple tests. To highlight the strongest associations, we also report the linear regression results using FDR setting the level of significance at .05.

## Results

Age, total brain volume, and sex did not differ between groups. Fewer patients (2 of 37) than control subjects (7 of 38) were left-handed, although this difference was not statistically significant. All cognitive scores were significantly worse in patients (at trend level for premorbid IQ; Table 1). Duration of treatment and DUP, positive, negative, and disorganization scores are shown on Table 1.

### Group Differences in Cortical Parameters

Analyses were performed with and without three patients with schizoaffective disorder. Results remained unchanged when the latter were excluded and findings for the whole group are reported (Table 2).

Whole-brain cortical thickness (vertex by vertex) did not differ between groups.

Cortical parameters in frontal and temporal regions: 1) Cortical thickness did not vary by diagnosis or sex. Age was more closely associated with thinning in the frontal than in the temporal cortex (regional mean difference in thinning per year = −.0085 mm/year; 95% confidence interval [CI] −.0113 to −.0057; *p* < .001], with thinning of .0073 mm/year (95% CI −.0109 to −.0038; *p* < .001) in the frontal region. 2) Cortical area was not related to age or side in frontal or temporal regions but was larger in male subjects irrespective of diagnosis (regional mean difference between males and females = 416.13 mm^2^; 95% CI 153.16 to 679.09); *p* = .002) and more so for the frontal (sex mean difference = 2679.29 mm^2^; 95% CI 1995.44 to 3363.13; *p* < .001) than the temporal regions (sex mean difference = 2263.16 mm^2^; 95% CI 1579.32 to 2947.01; *p* < .001). In patients, temporal cortical area was smaller than in controls (regional means difference in patients = −724.12 mm^2^; 95% CI –1369.56 to –78.69; *p* = .028), explained by the smaller area in the superior (95% CI –326.42 to –67.32; *p* = .003), middle (95% CI −291.31 to −32.21; *p* = .014) and inferior (95% CI −271.43 to −12.33; *p* = .032) temporal parcellations. FDR-corrected analysis showed a reduction in right superior temporal area in patients (mean difference between patients and control subjects = −206.74 mm^2^; 95% CI −354.87 to −58.62); *p* = .028). In a post hoc multiple linear regressions of cortical thickness in these three parcellations, adjusted for age and sex, thickness was only reduced in the right superior temporal parcellation (mean difference between patients and control subjects = −.0751 mm; 95% CI −.1363 to −.0139; *p* = .017). There were no significant differences in frontal cortical area between groups. 3) Temporal cortical volume was smaller in patients (regional mean difference in patients = −2183.94 mm^3^; 95% CI −4104.17 to −263.70; *p* = .026), explained by reductions in the volume of the superior (95% CI −1052.96 to −216.31; *p* = .003), middle (95% CI −936.38 to −99.73; *p* = .015), inferior temporal (95% CI −938.57 to −101.92; *p* = .015), and fusiform (95% CI −939.37 to −102.72; *p* = .015) parcellations. Left frontal cortical volume was larger than right (mean difference between sides = 740.47 mm^3^; 95% CI 42.03 to 1438.91; *p* = .038). There were no group differences in the temporal cortical volume (mean difference between sides = −218.41 mm^3^; 95% CI −916.85 to 480.03; *p* = .540). 4) Cortical parameters were not associated with duration of treatment or DUP or syndrome scores. In a post hoc analysis of parcellations with reduced cortical area, using linear regressions adjusted for DUP, age, and sex, a trend level association with treatment duration (area increase of 1.46 mm^2^/day of untreated psychosis; 95% CI −.04 to 2.97; *p* = .056) was present for the superior temporal parcellation.

### Cortical Parameters and Cognition

Whole group and the schizophrenia-only subgroup results are given for working memory because minor differences occurred when schizoaffective disorder patients were excluded.

#### Premorbid IQ

**Figure 2:**
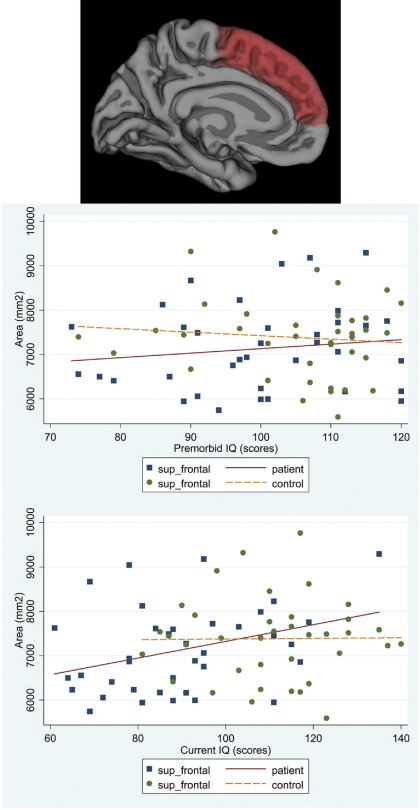
Scatter plot of the associations between premorbid IQ and current IQ with the average cortical area for the right and left hemispheres in patients and controls: superior frontal.

**Figure 3:**
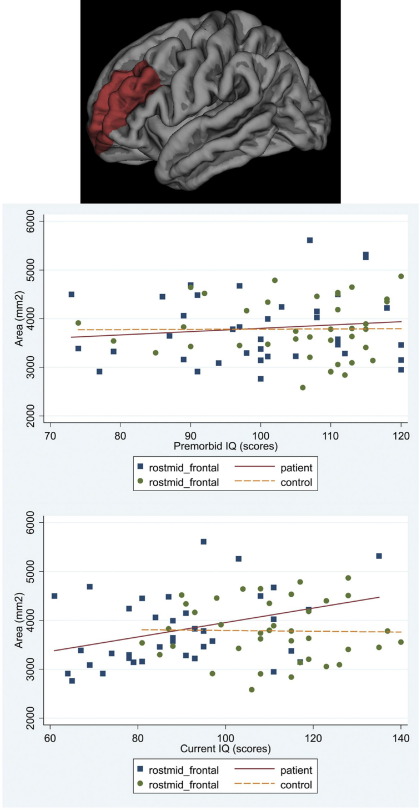
Scatter plot of the associations between premorbid IQ and current IQ with the average cortical area for the right and left hemispheres in patients and controls: rostral middle frontal.

**Figure 4:**
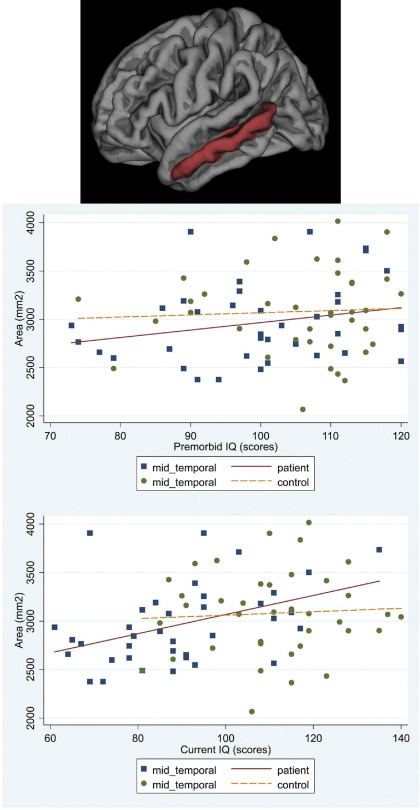
Scatter plot of the associations between premorbid IQ and current IQ with the average cortical area for the right and left hemispheres in patients and controls: middle temporal.

**Figure 5:**
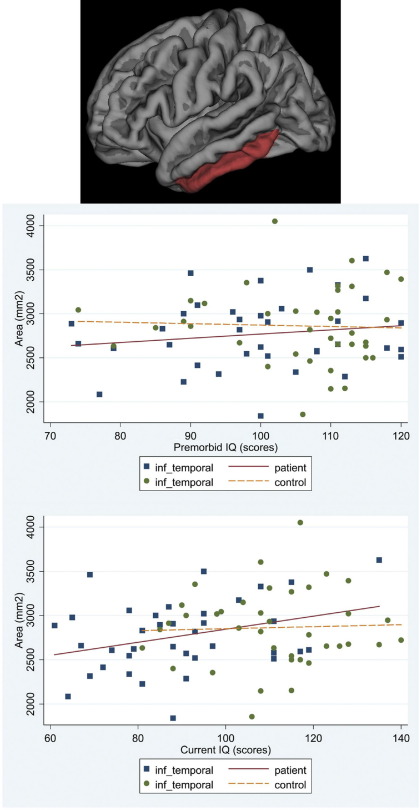
Scatter plot of the associations between premorbid IQ and current IQ with the average cortical area for the right and left hemispheres in patients and controls: inferior temporal.

**Figure 6:**
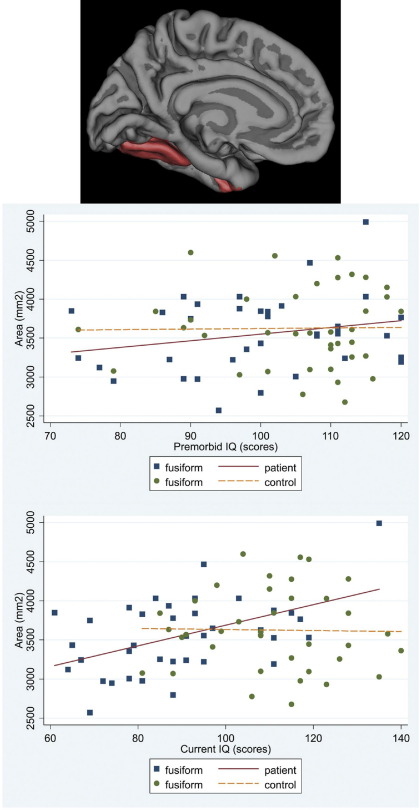
Scatter plot of the associations between premorbid IQ and current IQ with the average cortical area for the right and left hemispheres in patients and controls: fusiform parcellations.

In patients there was a significant association between cortical area and premorbid IQ for frontal and temporal regions (mean difference of regional area increase per IQ point = 6.04 mm^2^; 95% CI −8.35 to 20.43; *p* = .411). Higher premorbid IQ was associated with larger frontal cortical area (increase of 46.01 mm^2^ per premorbid IQ point; 95% CI 11.13 to 80.88; *p* = .010) accounted for by superior frontal parcellation (95% CI 2.91 to 23.05; *p* = .012). Higher premorbid IQ was associated with larger temporal cortical area (increase of 39.97 mm^2^; 95% CI 5.09 to 74.84; per IQ point; *p* = .025). The middle (95% CI 3.28 to 17.48; *p* = .004), inferior temporal (95% CI .41 to 14.61; *p* = .038), and fusiform (95% CI 4.19 to 18.39; *p* = .002) parcellations accounted for this association. No such associations were present in control subjects (Figures 2–6). No significant associations survived FDR correction.

In patients, there was a significant association between cortical volume and premorbid IQ for both frontal and temporal regions (mean difference of regional volume increase per IQ point = 26.80 mm^3^; 95% CI −27.26 to 80.87; *p* = .311). Higher premorbid IQ was associated with larger frontal cortical volume (increase of 151.87 mm^3^ per IQ point; 95% CI 48.91 to 254.84; *p* = .004) accounted for by the volumes of the superior (95% CI 23.40 to 81.83; *p* < .001) and rostral middle (95% CI 2.85 to 61.28; *p* = .031) parcellations. Higher premorbid IQ was also associated with temporal cortical volume (increase of 125.07 mm^3^ per IQ point; 95% CI 22.11 to 228.03; *p* = .017). The volumes in superior (95% CI 5.69 to 51.32; *p* = .014), middle (95% CI 12.61 to 58.25; *p* = .002), inferior (95% CI .36 to 45.99; *p* = .047), and fusiform (95% CI 13.94 to 59.58; *p* = .002) parcellations accounted for the association. These associations were not present in controls.

#### Current IQ

In patients, current IQ had a stronger association with frontal than temporal cortical area (mean difference of regional area increase per IQ point = 14.56 mm^2^; 95% CI 4.13 to 24.98; *p* = .006), with an increase of 44.93 mm^2^ per IQ point (95% CI 19.89 to 69.97; *p* < .001). The superior (95% CI 10.35 to 24.85; *p* < .001), rostral middle (95% CI 6.38 to 20.87; *p* < .001), and caudal middle (95% CI .04 to 14.54; *p* = .049) parcellations accounted for the association. There was an increase of 30.37 mm^2^ per IQ point (95% CI 5.33 to 55.41; *p* = .017) in the temporal cortical area. The middle (95% CI 3.87 to 14.17; *p* = .001), inferior (95% CI 1.43 to 11.73; *p* = .012) and fusiform (95% CI 7.17 to 17.48; *p* < .001) parcellations accounted for this association. Associations remained significant after FDR correction for the right fusiform (95% CI 6.23 to 21.89; *p* = .012) and rostral middle frontal (95% CI 5.27 to 23.86; *p* = .016) parcellations. No significant associations were found in controls (Figures 2–6).

In patients there was a significant association between cortical volume and current IQ for frontal and temporal regions (mean difference of regional volume increase per IQ point = 33.72 mm^3^; 95% CI −5.84 to 73.28; *p* = .095), with an increase of 131.99 mm^3^ of frontal cortical area per IQ point (95% CI 57.62 to 206.35; *p* = .001). The superior (95% CI 38.65 to 80.88; *p* < .001), rostral middle (95% CI 10.51 to 52.75; *p* = .003), and caudal middle (95% CI .60 to 42.84; *p* = .044) parcellations accounted for this association. Temporal cortical volume increased by 98.27 mm^3^ per IQ point (95% CI 23.90 to 172.63; *p* = .010) and the superior (95% CI 2.87 to 36.04; *p* = .022), middle (95% CI 13.41 to 46.59; *p* < .001, inferior (95% CI 3.98 to 37.15; *p* = .015), and fusiform (95% CI 22.49 to 55.66; *p* < .001) parcellations accounted for this association. No significant associations were present for controls.

#### Working Memory Span

In patients, there was a trend level association with larger frontal cortical area (281.43 mm^2^ increase in area per score point; 95% CI −13.83 to 576.68; *p* = .062) that reached significance when schizoaffective disorder patients were excluded (95% CI 8.99 to 696.42; *p* = .044).

In patients, there was a stronger association with frontal than temporal cortical volume (mean difference of regional volume increase per score point = 569.12 mm^3^; 95% CI 123.13 to 1015.11; *p* = .012), with an increase of 962.94 mm^3^ per score point (95% CI 86.24 to 1839.64; *p* = .031); the superior frontal parcellation accounted for this association (95% CI 260.72 to 744.35; *p* < .001). No significant associations were present for control subjects.

Cortical thickness was not associated with IQ or working memory span. Scores of planning, working memory manipulation, and Rey Auditory Verbal Learning Test were not significantly associated with cortical parameters. There were no significant associations by side for any cognitive variable.

## Discussion

Reduction in cortical volume, predominantly in temporal regions, due to smaller cortical area in patients with first-episode psychosis was our main finding, and area reductions were closely related to cognitive performance. Cortical thinning was only present in the right superior temporal region.

Our findings contrast with those of others reporting cortical thinning in patients with childhood (66,67), early-adulthood, or adult-onset (21,22) schizophrenia using SBM. The findings of Voets *et al.* (34), who reported reduced area and thickness in overlapping cortical regions, are closer to our own. These differences may be partly explained by the more severely compromised brain maturation trajectory in early onset schizophrenia (68,69). Methodologic variations may also be relevant. Thus, surface measurements in native space from the Desikan parcellations, used by Voets and colleagues (34) and ourselves, may be more sensitive than metric distortion used by others to estimate changes in cortical area.

The human brain is characterized by an expansion in the size and complexity of association areas in the neocortex (70), particularly prefrontal cortex (71), largely because of increased cortical area with little change in cortical thickness (72). In early fetal development, cortical area is determined by the migration of radial columns from the ventricular zone to the cortical plate (73,74), followed by the asymmetrical division of precursor cells in the ventricular zone and subsequent migration to the cortical plate increasing its thickness but not its area. Although neuronal migration is complete by the 25th week of gestation, glial migration and growth of cortical connections continue for longer with further increases in cortical surface, which is also influenced by differential expansion of cortical layers (75). Our finding of reduced area, without change in cortical thickness suggests a disruption of corticogenesis at a time of rapid cortical expansion, that is, late pregnancy and the perinatal period. Contemporary white matter abnormalities may have further reduced cortical area, as is thought to be the case in very low-birth-weight adolescents in whom area reduction is greater than cortical thinning (76,77). The reduction of brain volume over the first 2 decades of illness (78–80) suggests that mechanisms operating around disease onset may also be relevant. Regional gray matter changes may also be induced by atypical and typical antipsychotics (81), although we failed to find an association with treatment duration.

The pattern of cortical abnormalities reported here differs from that in degenerative conditions. Cortical thinning without area changes has been reported in early Huntington disease (18) and in Alzheimer's disease (85,86), validated at postmortem in the latter using stereologic methods (87).

We did not find significant correlations between cortical abnormalities and symptom severity, in keeping with other (82,83), but not all (84), SBM studies, but we found a strong association between IQ and frontotemporal cortical area. General intelligence depends on neural networks critically involving frontal and parietal cortex (88,89). It has high heritability and correlates strongly with gray matter volume (90,91) in normal twins (92) and singletons (93). These studies have mainly measured cortical thickness (94–96), which is modified by experience-dependent plasticity (97). Cognitive impairment, integral to schizophrenia (98), is best characterized by a generalized deficit (99,100). Those with schizophrenia have lower IQs than their childhood peers (101), and 40%–45% may decline further by illness onset (102–107). This is supported by the closer correlation between cortical area and current rather than premorbid IQ in our patients. We have previously reported that premorbid IQ and IQ at illness onset are prognostic indicators of clinical outcome 3 to 4 years later (106,108). The association between cortical area and IQ reported here suggests that cortical area changes may have prognostic relevance.

There are limitations to our study. Abnormalities in other than frontotemporal cortical areas cannot be excluded. It remains possible, although unlikely, that changes in cortical thickness could have been detected in a larger sample. However, Freesurfer reliability studies (109) suggest that differences in cortical thickness of less than .1 mm could have been detected with our sample size. Moreover, in a more detailed, regional analysis, cortical thinning was only present in one (the right superior temporal) of several temporal parcellations with reduced area suggesting that cortical abnormalities vary in different regions.

## Figures and Tables

**Table 1:** Demographic and Cognitive Measures in Patients and Controls

Variable Measured	Patients (*n* = 37)	Control Subjects (*n* = 38)	Comparison
Age (years)	26.8 (8.8) [16–49]	25.0 (5.4) [16–37]	*t*(73) = −1.06, *p* = .291
Male Sex, *n* (%)	25 (67.6)	22 (57.9)	χ^2^(1) = .75, *p* = .387
Left-Handed, *n* (%)	2 (5.4)	7 (18.4)	χ^2^(1) = 3.01, *p* = .083
Total Brain Volume (mm^3^)	1543685 (198405.4)	1571956 (188197.2)	*t*(73) = .63, *p* = .529
Premorbid IQ	99.8 (12.9) [73–120]	105.3 (11.2) [74–120]	*t*(74) = −1.99, *p* = .051
Current IQ	89.6 (17.6) [61–135]	110.6 (15.0) [81–140]	*t*(74) = −5.63, *p* < .001
Working Memory Span	5.5 (1.6) [2–9]	6.5 (1.2) [4–9]	*t*(74) = −3.24, *p* = .002
Planning	7.2 (2.7) (0−12)	8.8 (1.6) [6–12]	*t*(74) = −3.12, *p* = .003
Working Memory Manipulation	33.9 (16.7) (0−58)	15.4 (14.3) (0−60)	*t*(74) = 5.26, *p* < .001
Rey Auditory Verbal Learning Test	38 (8.4) [14–52]	49.4 (10.4) [18–64]	*t*(74) = −5.03, *p* < .001
Duration of Treatment (days)	80 (56) [9–186]	NA	NA
Duration of Untreated Psychosis (months)	10.4 (22.8) (0−126)	NA	NA
Factor 1, Negative Syndrome	.29 (.25) (0−.8)	NA	NA
Factor 2, Positive Syndrome	.75 (.20) (0−1)	NA	NA
Factor 3, Disorganization Syndrome	.27 (.25) (0−.8)	NA	NA

Values are means (SD) [range]. NA, not applicable.

**Table 2:** Cortical Parameters in Frontal and Temporal Regions in Patients and Controls Unadjusted by Age or Gender

Region of Cortex	Thickness (mm)^a^		Surface Area (mm^2^)^b^		Volume (mm^3^)^b^	
	Patients	Controls	Patients	Controls	Patients	Controls
Frontal						
Left	2.71 (.15)	2.70 (.13)	15680.59 (2086.42)	16048.87 (1989.18)	47014.73 (6395.26)	47862.50 (4818.93)
Right	2.73 (.15)	2.69 (.13)	15567.30 (2252.66)	15773.68 (1960.96)	46597.27 (6766.11)	46807.53 (4788.16)
Temporal						
Left	2.72 (.13)	2.71 (.11)	13782.35 (1557.68)	14275.03 (1803.94)	43358.57 (5253.97)	44940.76 (4817.45)
Right	2.73 (.15)	2.75 (.12)	13795.49 (1652.54)	14183.13 (1731.98)	43539.14 (5047.07)	45196.03 (4879.04)

a Values are means (SD).

b Values are means (SD) of the sums of six parcellations each.
